# The prognostic value of YAP1 on clinical outcomes in human cancers

**DOI:** 10.18632/aging.102358

**Published:** 2019-10-15

**Authors:** Ying Wu, Yanshen Hou, Peng Xu, Yujiao Deng, Kang Liu, Meng Wang, Tian Tian, Cong Dai, Na Li, Qian Hao, Dingli Song, Ling hui Zhou, Zhijun Dai

**Affiliations:** 1 Department of Breast Surgery, The First Affiliated Hospital, College of Medicine, Zhejiang University, Hangzhou, China; 2 Department of Oncology, The 2nd Affiliated Hospital of Xi’an Jiaotong University, Xi’an, Shaanxi, China; 3 Department of Anesthesiology, The 3rd Affiliated Teaching Hospital of Xinjiang Medical University (Affiliated Tumor Hospital), Urumqi, Xinjiang, China; 4 Department of Hepatobiliary Surgery, The First Affiliated Hospital of Xi'an Jiaotong University, Xi'an, Shaanxi, China

**Keywords:** YAP1, cancer, prognosis, meta-analysis

## Abstract

Background: As an important downstream factor in the Hippo pathway, yes-associated protein 1(YAP1) has been detected to be elevated in various cancers and demonstrated to play a role in tumor development. Therefore, we evaluated by a meta-analysis the prognostic value of YAP1 in cancer patients.

Results: Sixty-eight studies with 8631 patients were identified. The results indicated that YAP1 overexpression predicted unfavorable patient prognosis in studies with overall survival (OS) (HR=1.76, 95%CI: 1.50-2.06, *p*<0.001) and disease-free survival (DFS) (HR=1.39, 95%CI: 1.22-1.59, *p*<0.001), as well as in studies with recurrence-free survival (RFS) (HR=2.38, 95%CI: 1.73-3.27, *p*<0.001), and disease-specific survival (DSS) (HR=2.04, 95%CI: 1.55-2.70, *p*<0.001). Meanwhile, YAP1 overexpression was also observed to be significantly associated with worse OS in GEPIA (HR=1.2, *p*<0.001).

Conclusions: Overexpression of YAP1 showed great association with poorer prognosis in patients with various cancers, particularly liver cancer. Therefore, YAP1 might be an important prognostic marker and a novel target of cancer therapy.

Methods: We searched for potential publications in several online databases and retrieved relevant data. Overall and subgroup analyses were performed. Begg’s and Egger’s tests were used to assess publication bias. Online dataset GEPIA was used to generate the survival curves and verify the prognostic role of YAP1 in patients with tumors.

## INTRODUCTION

Cancer, a health challenge worldwide, is a dominating cause of death mainly in developed countries, with high incidence and low curative rate [1]. In 2018, there were 18.1 million people diagnosed with cancer and 9.6 million people died of cancer all over the world [2]. Until now, the general treatment includes surgery, chemotherapy, radiotherapy and the immune therapy, which is found to be effective in a multitude of cancers. Despite the dramatic advancements in medical treatment, there are still millions of people suffering from it.

Cancers are derived from the excessive growth of cancer cells, which activate the mechanisms to promote the cells growth and cancer progression [3]. A variety of studies had proved the important role of cellular signaling pathways in cancer initiation and development [4]. Hippo pathway, an important growth regulatory pathway, plays an integral role in controlling the size of organs and in the renewal of stem cells by regulating cell proliferation and apoptosis [4, 5]. The components of the Hippo pathway, including Warts, Salvador, mob-as-tumor suppressor, and Hippo, were first discovered in *Drosophila* by using genetic screens to search the tumor suppressor genes [6–15]. YAP1 is a transcription co-activator and main downstream effector of the Hippo pathway. Previously, YAP1 was thought to be an apoptosis-promoting protein. However, Huang et al. suggested that YAP was likely an oncogene rather than an apoptosis-promoting protein [16]. Meanwhile, some studies put forward that the function of YAP1 was different according to its location of expression. Nuclear YAP1 can promote cell growth and restrain apoptosis, whereas cytoplasmic YAP1 participate in cell apoptosis [16, 17]. Subsequently, many studies demonstrated that YAP1 was a negative prognostic marker in many tumors and promoted epithelial-mesenchymal transition, which indicated that YAP1 might induce cancer metastasis and invasion [18, 19]. In contrast, many researchers held opposite views. Matallanas et al. [20] reported that alleviating YAP cytoplasmic retention was related to p73-mediated apoptosis, indicating that YAP might play a role in tumor suppression. Consistent with it, Sun et al. [21] considered the cytoplasmic YAP expression was related with favorable prognosis.

Therefore, it is critical to investigate the relationship between YAP1 and cancer. The first meta-analysis regarding YAP1 was published three years ago, and reported that the positive expression of YAP1 was associated with poorer prognosis in various cancers [22]. In recent years, an increasing number of studies have been published which explored the correlation between YAP1 and patient prognosis with cancers. In this study, we gathered more studies to assess the prognostic value of YAP1 overexpression in patients with cancer.

## RESULTS

### Characteristics of studies included in the meta-analysis

**Figure 1:**
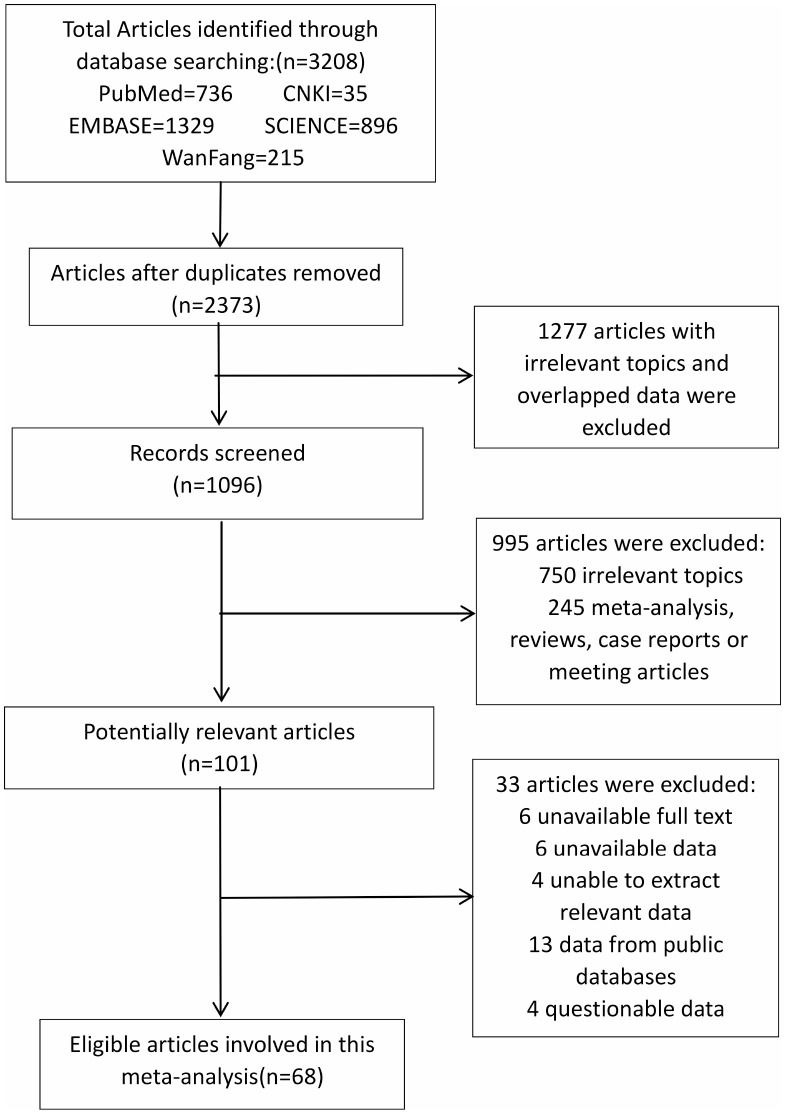
**The flow diagram of studies selection in this meta-analysis.**

In total, 3208 studies were searched initially, and 101 were considered potentially relevant articles. Among them, 33 studies were excluded (6 without full text, 6 with unavailable data, 4 in which we were unable to extract relevant data, 13 with data from databases, and 4 with questionable data). In the process of selecting literature, some studies were excluded due to the questionable data, such as the studies of Han et al., Fan Ye et al., and Wang et al. [23–25]. The confidence interval of the study conducted by Salcedo Allende et al. [26] was too narrow for the studied to be included in our study. Considering the same patients were possibly included indifferent public databases, we excluded the 13 studies that used results from public databases. Finally, 69 studies [18, 21, 27–92] that met our criteria were selected in our meta-analysis, and their characteristics were summarized in Supplementary Table 3. The flow diagram of the literature selection is shown in Figure 1. Besides, a scoring summary of all high-quality studies considered for this analysis were presented in Supplementary Table 2.

Of these 68 studies, 53 evaluated the relationship between YAP1 and OS, 19 for DFS, 7 for progression-free survival (PFS), 8 for RFS, and 5 for DSS. Collectively, these 68 included studies contained data for 8631 patients, from 12 countries, who were diagnosed with 18 different types of cancer, such as liver cancer, gastric cancer, and breast cancer, and so on.

### Meta-analysis results

**Figure 2:**
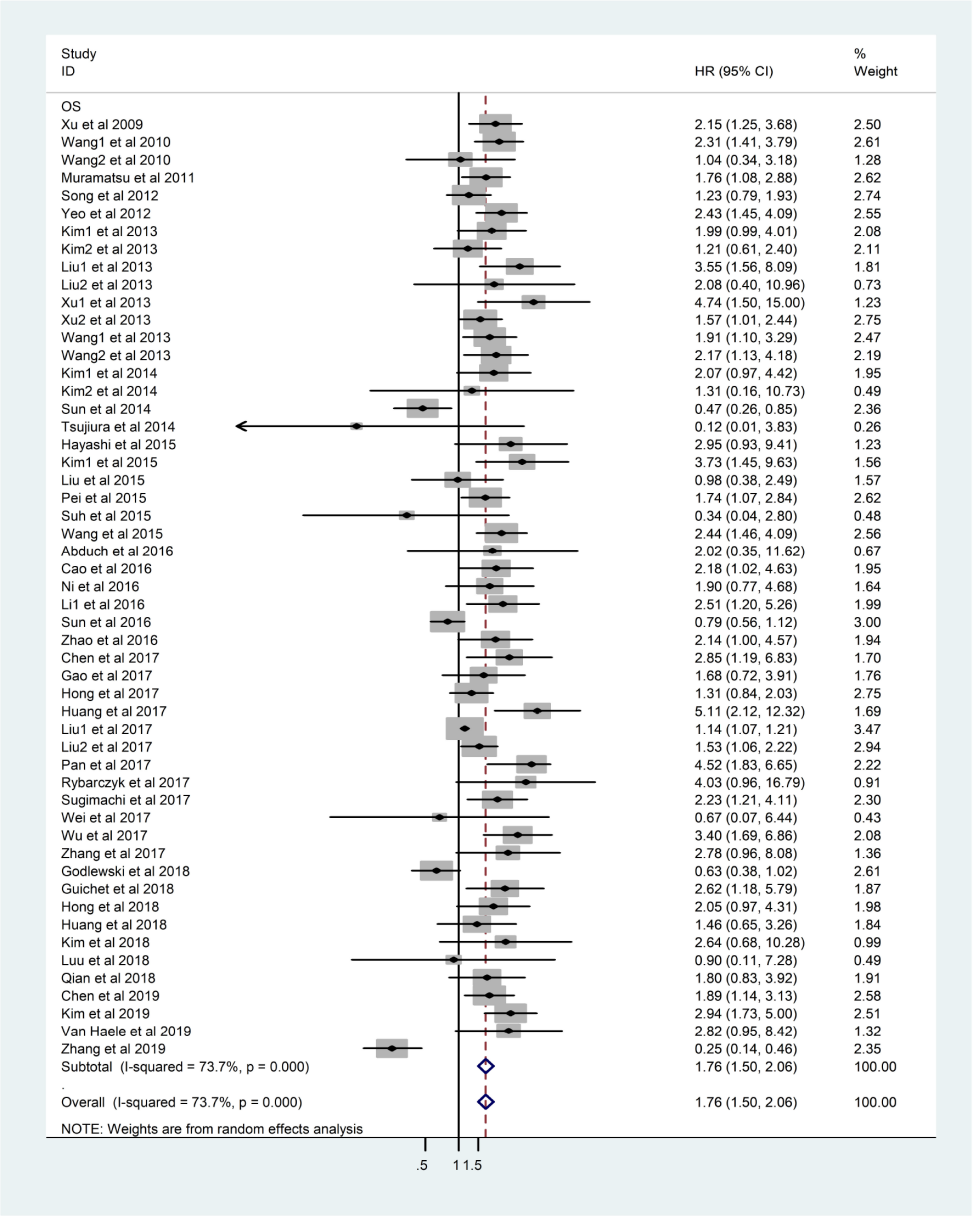
**Forest plot of HR for association between YAP1 overexpression and OS.** Note: Weights are from random-effects analysis. Abbreviations: CI confidence interval; HR hazard ratio; OS overall survival.

There was obvious heterogeneity of OS in 53 studies (I^2^=73.7%, *p*<0.001) and a random-effects model was applied. The result indicated that overexpression of YAP1 would lead to poorer OS (HR=1.76, 95%CI: 1.50-2.06, *p*<0.001) (Figure 2).

**Figure 3:**
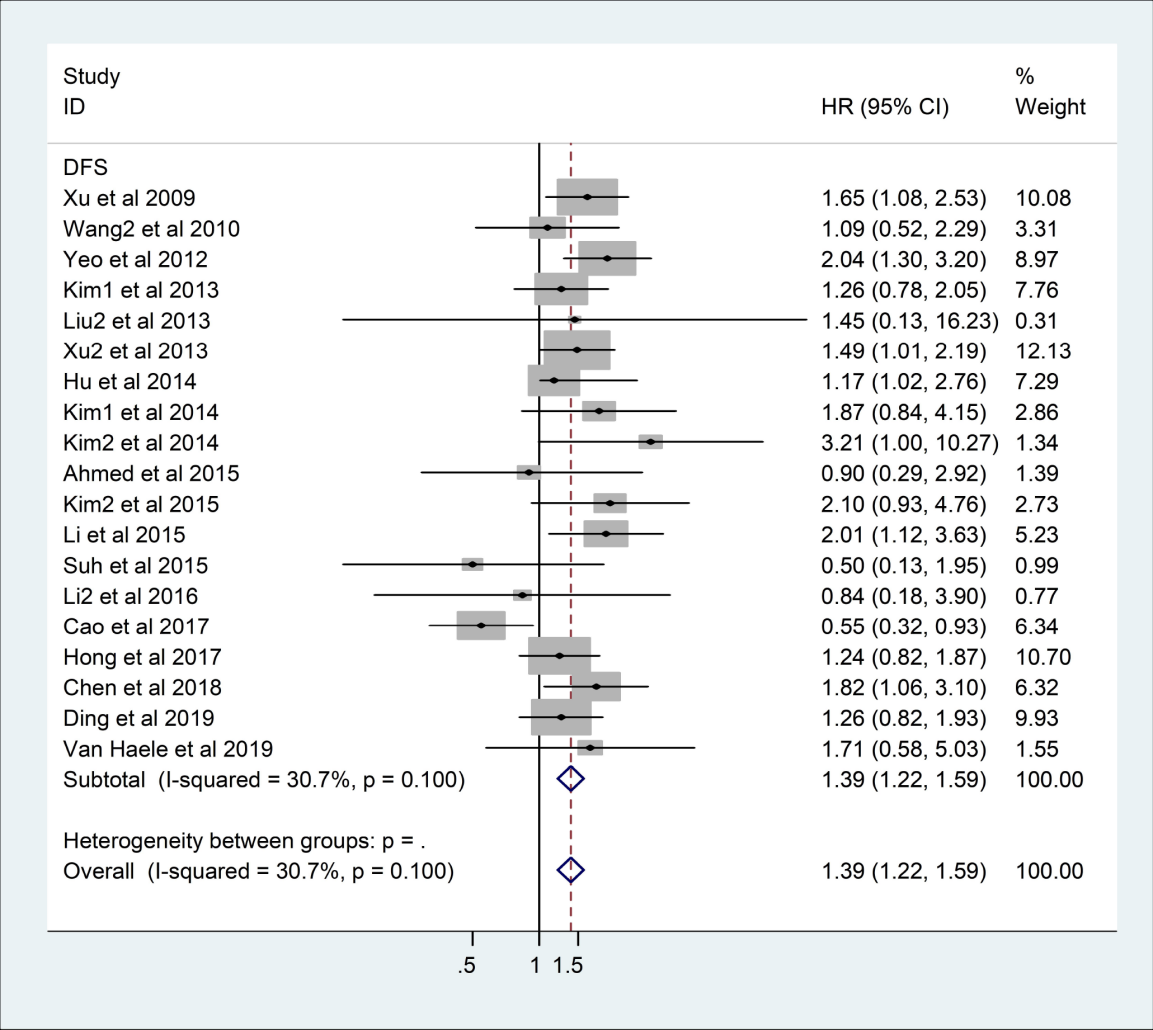
**Forest plot of HR for association between YAP1 overexpression and DFS.** Note: Weights are from fixed-effects analysis. Abbreviations: CI confidence interval; DFS disease-free survival; HR hazard ratio.

As the heterogeneity was not significant, the fixed-effects model was applied in 19 DFS studies comprising 3013 patients (I^2^=30.7%, *p*=0.10). The results showed that the overexpression of YAP1 was related to poorer DFS (HR=1.39, 95%CI: 1.22-1.59, *p*<0.001) (Figure 3).

As shown in Table 1, 7 studies with 652 patients, 8 studies with 954 patients, and 5 studies with 500 patients reported PFS, RFS, and DSS, respectively. Heterogeneity was found in PFS and RFS (PFS: I^2^=69.2%, *p*=0.003; RFS: I^2^=55.6%, *p*=0.027), so we used a random-effects model to evaluate the pooled HR and its 95% CI (PFS: HR=1.62, 95%CI: 0.96-2.73, *p*=0.069; RFS: HR=2.38, 95%CI: 1.73-3.27, *p*<0.001); for DSS, pooled HR (HR= 2.04, 95%CI: 1.55-2.70, *p*<0.001), for insignificant heterogeneity (I^2^=0%, *p*=0.82) was calculated using a fixed-effects model. The results suggested that YAP1 overexpression would predict poorer RFS and DSS, but not PFS (Supplementary Figure 1).

**Table 1:** Subgroup analysis of meta-analysis.

**Analysis**	**No of patients**	**No of studies**	**HR(95%CI)**	***P*-value**	**Heterogeneity**	
					***I*^2^(%)**	**P-value**
**OS**	6771	53	1.76(1.50-2.06)	**<0.001**	73.70%	<0.001
**Ethnicity**						
Asian	6271	46	1.78(1.51-2.10)	**<0.001**	74.90%	<0.001
Non-Asian	500	7	1.51(0.69-3.30)	0.304	66.70%	0.006
**Tumor types**						
Liver cancer	1273	11	1.93(1.62-2.31)	**<0.001**	0.00%	0.707
Lung cancer	546	5	1.54(0.85-2.82)	0.156	79.90%	0.001
Esophageal cancer	404	3	2.12(1.51-2.97)	**<0.001**	0.00%	0.590
Gastric cancer	1474	8	1.58(1.00-2.51)	0.051	77.30%	0.001
Colorectal cancer	838	7	1.48(0.75-2.90)	0.256	86.10%	<0.001
Bladder cancer	268	2	3.20(1.76-5.83)	**<0.001**	0.00%	0.719
Breast cancer	983	3	2.48(1.40-4.38)	**0.002**	0.00%	0.527
Ovarian cancer	236	3	1.49(0.89-2.49)	0.132	0.00%	0.571
Renal cancer	140	2	1.40(0.23-8.51)	0.712	82.70%	0.016
Others	609	9	1.69(0.975-2.93)	0.062	68.20%	0.001
**Method**						
IHC	6559	50	1.73(1.47-2.03)	**<0.001**	74.60%	<0.001
PCR	212	3	3.01(1.35-6.69)	**0.007**	0.00%	0.835
**Staining location**						
YAP1 expression	4131	34	1.90(1.54-2.33)	**<0.001**	76.90%	<0.001
Nuclear YAP1 expression	2516	19	1.63(1.29-2.07)	**<0.001**	51.20%	0.005
Cytoplasmic YAP1 expression	535	5	1.55(0.71-3.40)	0.275	81.00%	<0.001
**HR estimation**						
Univariate analysis	2774	31	1.78(1.45-2.17)	**<0.001**	65.80%	<0.001
Multivariate analysis	3997	22	1.77(1.32-2.37)	**<0.001**	78.90%	<0.001
**DFS**	3013	19	1.39(1.22-1.59)	**<0.001**	30.70%	0.100
**Ethnicity**						
China	1295	10	1.32(1.11-1.57)	**0.001**	43.90%	0.066
Korea	1605	7	1.54(1.22-1.93)	**<0.001**	26.50%	0.226
Others	113	2	1.26(0.57-2.77)	0.562	0.00%	0.427
**Tumor types**						
Liver cancer	856	6	1.59(1.29-1.95)	**<0.001**	0.00%	0.864
Gastric cancer	573	4	1.15(0.86-1.52)	0.352	0.00%	0.658
Breast cancer	1155	4	1.09(0.81-1.47)	0.560	74.70%	0.008
Others	429	5	1.79(1.25-2.56)	**0.001**	0.00%	0.589
**Staining location**						
YAP1 expression	1818	14	1.32(1.13-1.54)	**0.001**	37.90%	0.074
Nuclear YAP1 expression	1324	7	1.67(1.32-2.12)	**<0.001**	16.90%	0.301
**HR estimation**						
Univariate analysis	1188	11	1.40(1.17-1.69)	**<0.001**	0.00%	0.537
Multivariate analysis	1825	8	1.38(1.13-1.68)	**0.001**	58.90%	0.017
**PFS**	652	6	1.62(0.96-2.73)	0.069	69.20%	0.003
**RFS**	954	8	2.38(1.73-3.27)	**<0.001**	55.60%	0.027
**DSS**	500	5	2.04(1.55-2.70)	**<0.001**	0.00%	0.820

Abbreviations: *CI* confidence interval; *DFS* disease-free survival; *DSS* disease-specific survival; *HR* hazard ratio; *IHC* immunohistochemistry; *M* multivariate; *NA* no applicable; *OS* overall survival; *PFS* progression-free survival; *RFS* recurrence-free survival; *U* univariate.

### Subgroup analysis of OS

To explore the source of heterogeneity, we further carried out subgroup analysis according to four possible factors (ethnicity, tumor type, method, staining location and analysis types of studies), summarized in Table 1. The subgroup analysis by ethnicity suggested that overexpression of YAP1 was a negative prognosticator in Asian patients (HR=1.78, 95%CI: 1.51-2.10, *p*<0.001), but not in non-Asian patients. In the tumor type subgroup, significant associations were found in liver cancer (HR=1.93, 95%CI: 1.62-2.31, *p*<0.001), esophageal cancer (HR=2.12, 95%CI: 1.51-2.97, *p*<0.001), breast cancer (HR=2.48, 95%CI: 1.40-4.38, *p*=0.002), and bladder cancer (HR=3.20, 95%CI: 1.76-5.83, *p*<0.001). When stratified by method, IHC (HR=1.73, 95%CI: 1.47-2.03, *p*<0.001) and PCR (HR=3.01, 95%CI: 1.35-6.69, *p*=0.007), both showed a significant relationship with poorer OS. When analyzed by YAP1 staining location, total YAP1 expression and nuclear YAP1 expression showed great significance with dismal prognosis in tumor patients (YAP1 expression: HR= 1.90, 95%CI: 1.54-2.33, *p*<0.001; nuclear YAP1 expression: HR=1.63, 95%CI: 1.29-2.07, *p*<0.001). Regarding the type of analysis, univariate analysis (HR=1.78, 95%CI: 1.45-2.17, *p*<0.001) indicated that overexpression of YAP1 was significantly related with elevated OS, as did the multivariate analysis (HR=1.77, 95%CI: 1.32-2.37, *p*<0.001).

### Subgroup analysis of DFS

Based on ethnicity, tumor type, staining location, and analysis type, subgroup analysis was also performed in the studies reporting DFS. YAP1 overexpression in Korean studies (HR=1.54, 95%CI: 1.22-1.93, *p*<0.001), as well as in Chinese (HR=1.32, 95%CI: 1.11-1.57, *p*=0.001), was associated with poor DFS. In the subgroup analysis by tumor type, a significant relationship was found between patients with liver cancer (HR=1.59, 95%CI: 1.29-1.95, *p*<0.001) and DFS. Regarding staining location, subgroup analysis suggested that both total YAP1 expression (HR=1.32, 95%CI: 1.13-1.54, *p*=0.001) and nuclear YAP1 expression (HR=1.67, 95%CI: 1.32-2.12, *p*<0.001) were predictors of poorer DFS. According to the subgroup result of analysis type, univariate analysis (HR=1.40, 95%CI: 1.17-1.69, *p*<0.001) and multivariate analysis (HR=1.38, 95%CI: 1.13-1.68, *p*=0.001) showed significant associations with poorer DFS.

### Sensitivity analysis

**Figure 4:**
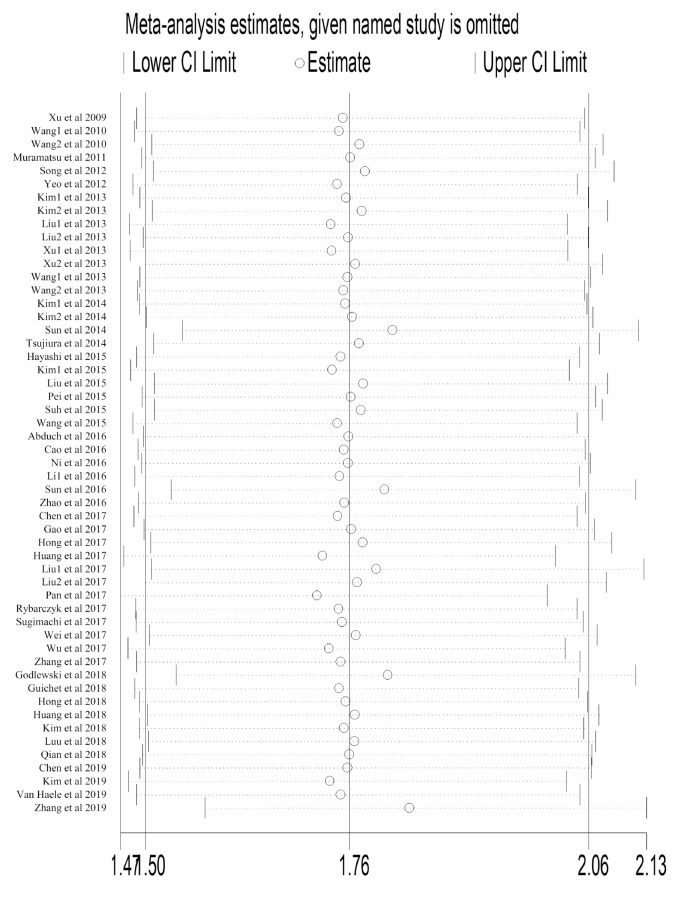
**Sensitivity analysis to evaluate the influence of every study reporting OS in our meta-analysis.** Abbreviation: OS overall survival; CI confidence interval.

Each study was omitted consecutively to explore its impact on the entire result. As shown in Figure 4, no individual study substantially altered the pooled HR of studies reporting OS, which implied the result was stable. Same result was observed in studies reporting DFS (Supplementary Figure 2).

### Publication bias

**Figure 5:**
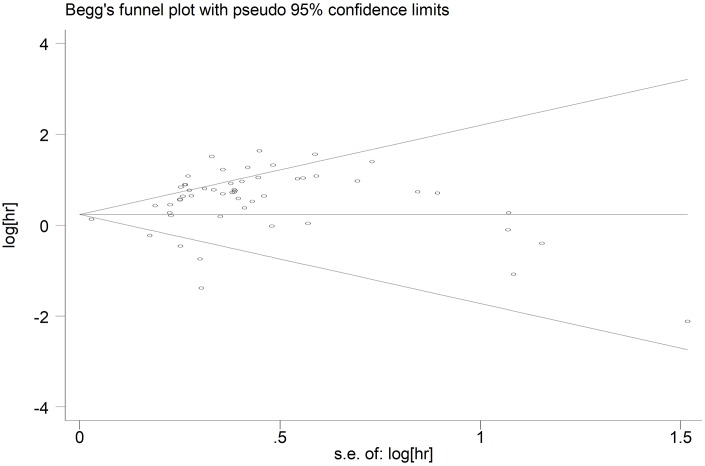
**Begg’s funnel plot of publication bias for included studies reporting OS.** Abbreviations: HR hazard ratio; OS overall analysis; SE standard error.

We generated the funnel plot via Begg’s test and investigated the potential publication bias using Egger’s test. As shown in the funnel plot of OS, the points which represented the results of each study distributed asymmetrically on both sides of the axis (Figure 5). However, publication bias was not found according to Begg’s test (*p*=0.395), but there was significant bias per Egger’s test (*p*<0.05). Therefore, considering the efficiency of two tests, we believed that there was publication bias among studies reporting OS. Publication bias was not detected in studies reporting DFS (Begg’s test: *p*=0.675; Egger’s test: *p*=0.812) (Supplementary Figure 3).

**Figure 6:**
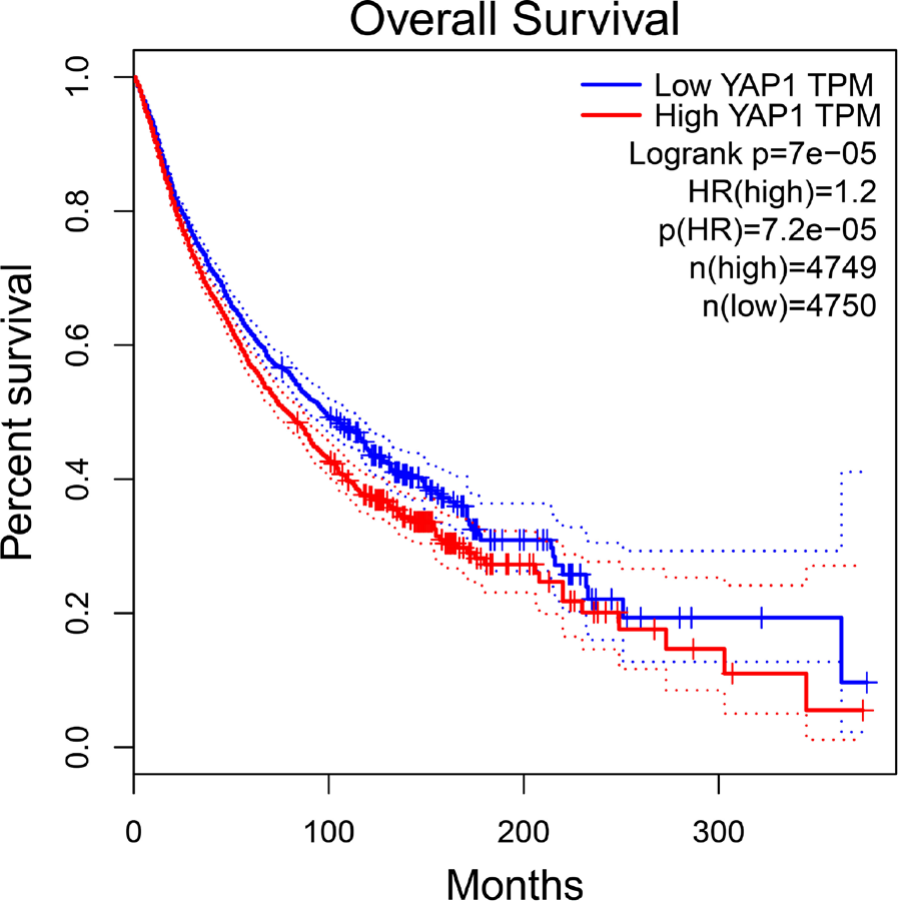
**The prognostic value of YAP1 in total patients with tumors in GEPIA. Abbreviations: HR hazard ratio.**

### Prognostic analysis in GEPIA

To further investigate the association between YAP1 expression and patients’ prognosis in various cancers, we performed the survival analysis using GEPIA. The results showed there was a significant relation between YAP1 overexpression and worse OS in total patients with tumors (HR=1.2, *p*<0.001) (Figure 6). However, no significance was observed between YAP1 overexpression and DFS in total patients with tumors. Furthermore, we found YAP1 overexpression might indicate unfavorable OS and DFS in patients with glioma, as well as worse OS in esophageal carcinoma patients and better DFS in bladder urothelial carcinoma patients. The results were described in Table 2.

**Table 2:** Association between YAP1 and cancer patients’ prognosis in GEPIA.

**Tumor type**	**No of YAP1 expression**		**OS**			**DFS**		
	**High**	**Low**	***p***	**HR(high)**	***p*(HR)**	***p***	**HR(high)**	***p*(HR)**
Total tumor	4746	4746	**0.00007**	1.2	0.000072	0.21	1	0.21
Liver cancer	199	199	0.2	1.2	0.2	0.67	1.1	0.67
Lung cancer	481	481	0.39	1.1	0.39	0.67	1.1	0.67
Esophageal carcinoma	91	91	**0.012**	0.56	0.013	0.96	1	0.95
Stomach adenocarcinoma	192	192	0.92	0.98	0.92	0.35	0.83	0.34
Colorectal cancer	181	180	0.095	0.69	0.096	0.79	1.1	0.79
Bladder Urothelial Carcinoma	201	201	0.14	1.2	0.14	**0.036**	1.4	0.037
Renal cancer	432	432	0.69	0.95	0.69	0.94	0.99	0.94
Breast invasive carcinoma	534	535	0.17	1.3	0.17	0.48	0.88	0.49
Uterine cancer	261	261	0.31	1.2	0.3	0.92	0.66	0.65
Ovarian serous cystadenocarcinoma	212	212	0.56	1.1	0.56	0.62	1.1	0.61
Glioma	338	338	**0.000002**	1.8	0.0000027	**0.00016**	1.6	0.00018
Others	1624	1624	**0.0012**	1.3	0.0012	0.069	1.1	0.069

Abbreviations: *DFS* disease-free survival; *HR* hazard ratio; *OS* overall survival.

## DISCUSSION

The mechanism involved in the development of cancer is complicated and is generally thought to be related to the dysfunction of cellular signaling pathways. Previous studies suggested the cellular signaling pathways could regulate the growth factors and expression of some vital genes that are associated with cell proliferation, migration, metabolism and junction between cells and so forth [93–95]. Hippo/YAP1 pathway was firstly discovered through gene screen of tumor suppressors in Drosophila. However, with the identifications of Hippo pathway components, it was demonstrated that Hippo/YAP1 pathway could control the organ size by regulating the balance between cell proliferation and apoptosis in mammalian [96]. Therefore, the dysfunction of Hippo/YAP1 pathway could imbalance the regulation, which could cause the cancer initiation. YAP1, as the main effector of Hippo pathway, plays a critical role in the development of cancers, standing at the cross point of different signaling pathways, mediated by upstream effectors and then regulating targets in collaboration with other transcription factors. Several important signaling pathways are known to participate in the activity of the Hippo/YAP1 pathway, these include TGF-β/SMAD, Wnt-β-catenin, Jak/Stat, Notch signaling pathway [97–100]. When crosstalking with the Sonic Hedgehog pathway, YAP1 can behave like an oncogene promoting cell proliferation and inhibiting cell differentiation [101]. However, in other studies, where p73-mediated cell apoptosis was recognized, YAP1 functioned as a tumor suppressor in response to DNA damage [102, 103].

In our study, overall and subgroup analyses were conducted to provide evidence of the association between the overexpression of YAP1 and patients’ prognosis. The results showed that overexpression of YAP1 predicted poorer OS, DFS, RFS, and DSS. The sensitivity analysis showed that no single study changed the pooled results significantly, which indicated the results of sensitivity analysis were stable. Subgroup analysis was conducted by ethnicity, tumor type, method, staining location, and HR estimation analysis type. According to the results of the subgroup analysis, there were no clear relations between YAP1 with OS in non-Asians, colorectal cancer, lung cancer, renal cancer, and ovarian cancer.

To clarify it further, we investigated the association between YAP1 expression and cancer patients’ prognosis in the online database, GEPIA. The results showed YAP1 expression was observed to be significantly related with worse OS, but not with DFS. Additionally, the same correlations were found between YAP1 expression and unfavorable OS and DFS in patients with glioma, as well as shorter OS in esophageal carcinoma patients and better DFS in bladder urothelial carcinoma patients. The differences between this meta-analysis and the online dataset might come from the following reasons. First of all, the detecting methods are thought to be the main reason for the differences. In most of the studies included in our meta-analysis, IHC was used to detect the expression of YAP1 protein, whereas the data of the online dataset were from the results of RNA sequencing. What’s more, in some specific cancer types, the number of patients that included in meta-analysis was much larger than the dataset. For example, although we have summed up the data of cholangio carcinoma and liver hepatocellular carcinoma, the total number of patients with liver cancer studied in the meta-analysis (1629), far exceed the number of the liver cancer patients represented in the online dataset (398). In addition, the cancer types are different. For instance, the breast cancer patients included in our meta-analysis represented various breast cancer subtypes, while the data in GEPIA included data for breast invasive carcinoma (BRCA) only. Therefore, while GEPIA provides valuable insights into gene-specific cancer survival outcomes, it is still being developed, and results obtained from the robust meta-analysis remain more convincing.

In the present study, we identified 14 studies that reported the relationship between prognosis of patients with liver cancer and YAP1 overexpression. Our results suggested that YAP1 overexpression predicted poorer OS and DFS. YAP1 was reported to be amplified and overexpressed in many cancers, and patients were suggested to receive further treatment, such as chemotherapy and hepatectomy. Although tumors can be removed by hepatectomy, rapid repair of the liver is crucial after partial hepatectomy, in which YAP1 increases and participates in the promotion of cell proliferation [104, 105]. The hedgehog pathway might be involved in this process. After blocking the hedgehog pathway in hepatic stellate cells, nuclear accumulation of YAP1 in neighboring hepatocytes did not occur [106]. Combining all these results of other studies and ours, we believe that YAP1 might induce unfavorable prognosis and be an important target in the treatment of liver cancer.

It is widely accepted that YAP1 is phosphorylated and accumulates in the cytoplasm such that it loses its ability to activate genes that promote cell invasion and proliferation and suppresses cell apoptosis once the Hippo pathway is activated [17, 107]. Therefore, to clarify the mechanisms of YAP1 functioning in cancer, we explored the association between staining location of YAP1 and cancer patients’ prognosis. We found total YAP1 expression and nuclear YAP1 expression indicated the unfavorable prognosis, both with OS and DFS, whereas the YAP1 expressed in cytoplasm was not related with cancer patients’ prognosis. Meanwhile, lots of studies reported that YAP1 expressed stronger in nuclei, while it was weaker in cytoplasm. The results might suggest nuclear YAP1 plays an important role in cancer, but it still needs more studies to be demonstrated furthermore.

Previously, Sun et al. [22] performed a meta-analysis, including 21 studies with 2983 patients, and reported that overexpression of YAP1 was associated with worse OS and DFS. Same results were demonstrated in the previous meta-analysis by Zhang et al. in gastrointestinal cancer. Compared with these studies, our study had some further research results. Firstly, our meta-analysis included a larger number of studies which were the latest. Secondly, in this study, not only OS and DFS, but RFS, PFS, and DSS were analyzed to understand the issue more comprehensively. Thirdly, having benefited from more studies, our study further explored the prognostic value of YAP1 expression in different specific cancers. Fourthly, we tried our best to illustrate the potential mechanism of YAP1 functioned in cancer development through subgroup analysis of YAP1 staining location.

Despite our efforts to make an accurate and comprehensive analysis, a few limitations inevitably existed in our study. First of all, some studies did not report clinical data; we had to extract the HR from the Kaplan-Meier survival curves indirectly, which might influence the accuracy of the original data. Secondly, publication bias was observed in our study among the studies reporting OS and we thought it was associated with the studies we included. Most of the studies on the relation between YAP1 and prognosis were based on Asian patients. Meanwhile, the bias might also be due to the non-publishing of studies with negative results. More studies are needed to resolve this limitation. Thirdly, significant heterogeneity between studies with OS, PFS, and RFS were detected. However, we failed to identify the source of heterogeneity by subgroup analysis. Then we minimized the effect by applying a random-effects model. Possibly, it was due to the differences in the methods of surgery and treatment, and baseline YAP1 expression level. Additionally, owing to the lack of clinical data, we could not confirm their contribution to heterogeneity.

## CONCLUSIONS

Overall, our study provides evidence that overexpression of YAP1 is significantly associated with the progression and recurrence of tumors and it might be a useful prognosticator in various cancers. However, future prospective studies with more complete and available clinical information are expected and required to confirm the results.

## METHODS

### Literature search strategy

Studies related to YAP1 were searched through PubMed, EMBASE, Web of Science, WANFANG, and CNKI. The main keywords used in the search were as follows: “Yes-associated protein 1”, “YAP1”, “Hippo”, “Neoplasms”, “Neoplasia”, “Tumor”, “Cancer”, “Malignancy”, “Prognosis”, “Survival” and “Metastasis”. The search strategy for PubMed is shown in Supplementary Table 1.

### Study selection criteria

Studies were included when they met the following criteria: (1) studies explored the association between YAP1 expression and patients’ prognosis; (2) studies declared the number and information of patients; (3) studies measured positive or high YAP1 expression in patients with any carcinoma by immunohistochemistry and polymerase chain reaction; (4) the endpoints were listed which are OS, RFS, DFS, PFS, and DSS; (5) the HRs with their 95% CIs or Kaplan-Meier survival curves were provided; (6) as for the overlapping data, the latest and most complete studies were included.

Studies were excluded if they: (1) were published meta-analyses, letters, abstracts, reviews or case reports; (2) did not provide survival information; (3) the data were not related to humans; (4) used data from public databases; or (5) were questionable data or the full text was not found.

### Data extraction

The information extracted from the included studies were: first author, publication year, patient source, number of patients, tumor type, specimen, methods, YAP1 expression, staining location, median follow-up time, prognostic outcome, HR estimation (when both univariate and multivariate analysis were provided, we included the outcomes of the multivariate analysis). We applied the HR with its 95% CI from the text directly or calculated the HR using Engauge Digitizer 4.0 by Tierney et al. through Kaplan-Meier survival curve analysis. The literature and data were selected and abstracted by two authors, and divergence views were resolved in a group meeting.

### Quality assessment

The NOS was used to assess the quality of the literature. Two authors of our group scored all eligible studies independently, and all discrepancies were discussed to arrive at a conclusion. The scores of studies included ranged from 6 to 8, which implied high quality.

### Prognostic analysis in GEPIA

Gene Expression Profiling Interactive Analysis (GEPIA) is a new online dataset, including 9736 tumors and 8587 normal samples from The Cancer Genome Atlas (TCGA) and Genotype-expression (GTEx) projects. In this study, for further verifying the association between YAP1 and patients’ prognosis with tumors, the GEPIA was used to perform the survival analysis.

### Statistical analysis

HRs and their 95% CIs were used to assess the relation between YAP1 expression and prognosis of patients with carcinoma. We evaluated the heterogeneity using the Q test and I^2^ test. Significant heterogeneity was defined by a p value <0.1 or I^2^ value >50%, based on which we applied a random-effects model; if no significant heterogeneity was found, we used a fixed-effects model. Subgroup analysis was conducted according to ethnicity, tumor type, staining location, and analysis type. In the sensitivity analysis, to estimate the impact of each study on our meta-analysis, we omitted individual studies consecutively. Begg’s test and Egger’s test were performed to assess the potential publication bias. If the funnel plots showed the points distributed asymmetrically on both sides of the axis, the publish bias was regarded to be significant. All statistical tests were bi-directional and a p-value less than 0.05 indicated statistical significance. Data analysis was performed using STATA 12.0 (Stata Corporation, College Station, Texas, USA).

## Supplementary Material

Supplementary Figures

Supplementary Table 1

Supplementary Table 2

Supplementary Table 3
