# Supplementary material for: The prognostic value of YAP1 on clinical outcomes in human cancers
Source: Aging (Albany NY). 2019 Oct 15;11(19):8681–700. doi: 10.18632/aging.102358 (PMC6814621; doi:10.18632/aging.102358)
Supplement: Supplementary Figures [file aging-11-102358-s004.pdf]

SUPPLEMENTARY FIGURES

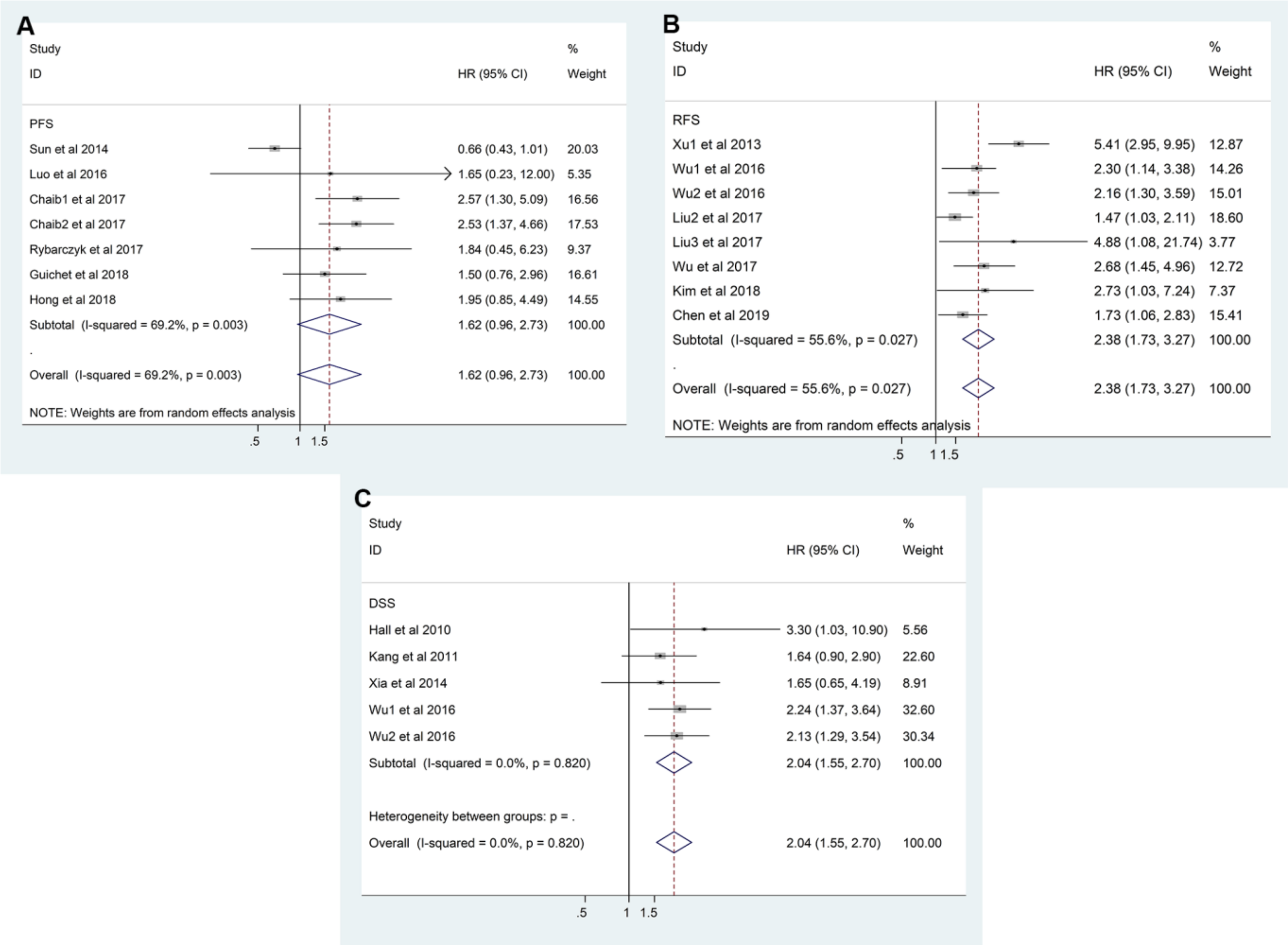

Supplementary Figure 1. Forest plot of HR for association between YAP1 overexpression and PFS, RFS and DSS. Note: Weights are all from random-effects analysis. Abbreviations: CI confidence interval; DSS disease-specific survival; HR hazard ratio; PFS progression-free survival; RFS recurrence-free survival.

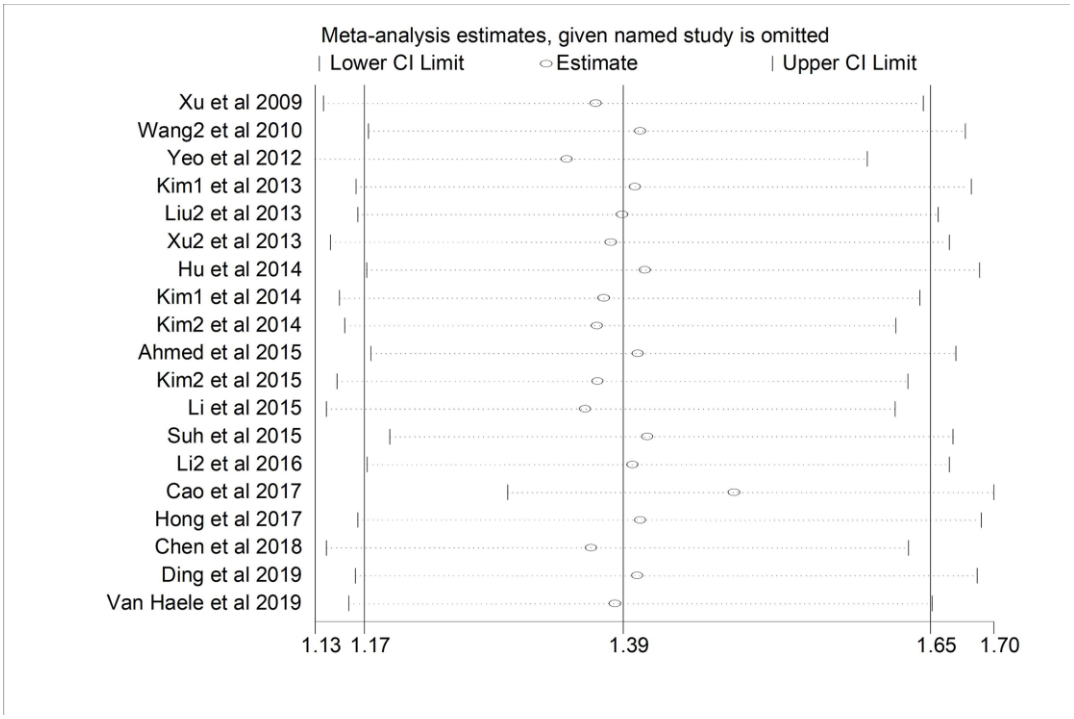

Supplementary Figure 2. Sensitivity analysis to evaluate the influence of every study reporting DFS in our meta-analysis. Abbreviation: DFS disease-free survival; CI confidence interval.

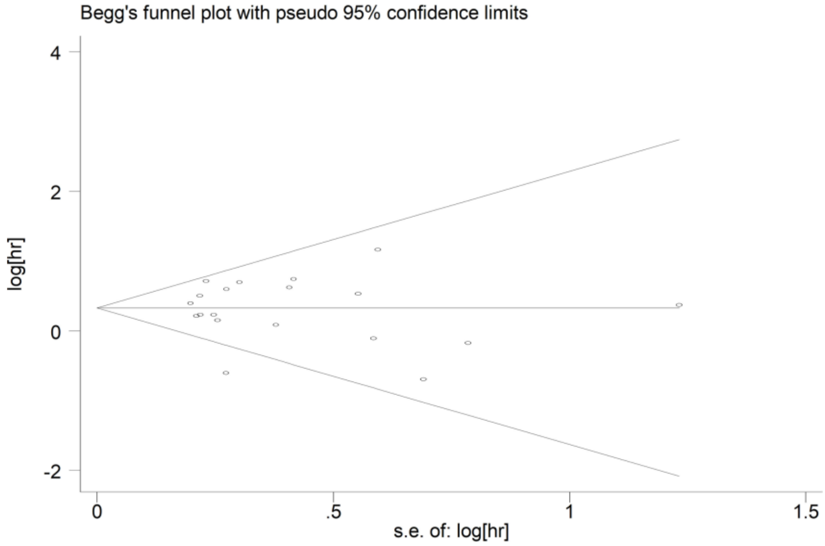

Supplementary Figure 3. Begg's funnel plot of publication bias for included studies reporting DFS. Abbreviation: DFS disease-free survival; CI confidence interval.
