# Supplementary material for: The prognostic value of YAP1 on clinical outcomes in human cancers
Source: Aging (Albany NY). 2019 Oct 15;11(19):8681–700. doi: 10.18632/aging.102358 (PMC6814621; doi:10.18632/aging.102358)
Supplement: Supplementary Table 1 [file aging-11-102358-s002.pdf]

Supplementary Table 1. The search strategy for PubMed.

**#1**	**“yes-associated protein 1” [Supplementary Concept]**
**#2**	YAP1 OR hippo
**#3**	#1 or #2
**#4**	“Neoplasms”[Mesh]
**#5**	(((((((Neoplasia) OR Neoplasias) OR Tumor) OR Tumors) OR Cancer) OR Cancers) OR Malignancy) OR Malignancies
**#6**	#4 or #5
**#7**	“Prognosis”[Mesh]
**#8**	(((Prognoses) OR Prognostic) OR prognosis) OR survival
**#9**	#7 or #8
**#10**	#3 and #6 and #9

Abbreviations: YAP1 yes-associated protein 1.
