# Supplementary material for: The prognostic value of YAP1 on clinical outcomes in human cancers
Source: Aging (Albany NY). 2019 Oct 15;11(19):8681–700. doi: 10.18632/aging.102358 (PMC6814621; doi:10.18632/aging.102358)
Supplement: Supplementary Table 2 [file aging-11-102358-s001.docx]

**Supplementary Table 2. NOS score of included studies.**

| First author | Representativeness of the exposed cohort | Selection of non exposed cohort | Ascertainment of exposure | Demonstration that outcome of interest was not present at start of the study | Comparability of cohorts on the basis of the design or analysis | Assessment of outcome | Was follow-up long enough for outcomes to occur | Adequacy of follow up of cohorts | NOS score |
| --- | --- | --- | --- | --- | --- | --- | --- | --- | --- |
|  | a.truly representative of the average in the community * | a.drawn from the same community as the export cohort * | a.secure record (eg. surgical record) * | a.yes * | a.study controls for YAP1 negative or low expression * | a.independent blind assessment * | a.yes * | a.complete follow up-all subjects accounted for * |  |
|  | b.somewhat representative of average in the community * | b.drawn from the different source | b.structured interview | b.no | b.study controls for other factors * | b.record linkage * | b.no | b.subjects lost to follow up unlikely introduce bias-small number lost＞20% follow up, or description provided of those lost) * |  |
|  | c.selected group of users eg. nurses, volunteers | c.no description of derivation of the non exposed cohort | c.written self report |  |  | c.self report |  | c.follow up rate＜20% and no description provided of those lost) |  |
|  | d.no description of derivation of the cohort |  | d.no description |  |  | d.no description |  | d.no statement |  |
| Xu et al | * | * | * | * | * |  | * | * | 6 |
| Hall et al | * | * | * | * | * |  | * |  | 6 |
| Wang^1^ et al | * | * | * | * | * | * | * |  | 7 |
| Wang^2^ et al | * | * | * | * | * | * | * | * | 8 |
| Kang et al | * | * | * | * | * |  | * |  | 6 |
| Muramatsu et al | * | * | * | * | * | * | * |  | 7 |
| Song et al | * | * | * | * | * | * | * |  | 7 |
| Yeo et al | * | * | * | * | * | * | * |  | 7 |
| Kim^1^ et al | * | * | * | * | * | * | * | * | 8 |
| Kim^2^ et al | * | * | * | * | * |  | * |  | 6 |
| Liu^1^ et al | * | * | * | * | * | * | * |  | 7 |
| Liu^2^ et al | * | * | * | * | * | * | * |  | 7 |
| Xu^1^ et al | * | * | * | * | * |  | * |  | 6 |
| Xu^2^ et al | * | * | * | * | * |  | * |  | 6 |
| Wang et al | * | * | * | * | * |  | * |  | 6 |
| Hu et al | * | * | * | * | * | * | * | * | 7 |
| Kim^1^ et al | * | * | * | * | * |  | * |  | 6 |
| Kim^2^ et al | * | * | * | * | * |  | * |  | 6 |
| Sun et al | * | * | * | * | * | * | * |  | 7 |
| Tsujiura et al | * | * | * | * | * | * | * |  | 7 |
| Xia et al | * | * | * | * | * |  | * |  | 6 |
| Ahmed et al | * | * | * | * | * | * | * |  | 7 |
| Hayashi et al | * | * | * | * | * | * | * |  | 7 |
| Kim^1^ et al | * | * | * | * | * | * | * |  | 7 |
| Kim^2^ et al | * | * | * | * | * |  | * |  | 6 |
| Li et al | * | * | * | * | * | * | * |  | 7 |
| Liu et al | * | * | * | * | * |  | * |  | 6 |
| Pei et al | * | * | * | * | * | * | * |  | 7 |
| Suh et al | * | * | * |  | * | * | * |  | 6 |
| Wang^1^ et al | * | * | * | * | * | * | * |  | 7 |
| Wang^2^ et al | * | * | * | * | * | * | * | * | 8 |
| Abduch et al | * | * | * | * | * | * | * |  | 7 |
| Ni et al | * | * | * | * | * |  | * |  | 6 |
| Li^1^ et al | * | * | * | * | * |  | * | * | 7 |
| Li^2^ et al | * | * | * | * | * | * |  | * | 7 |
| Luo et al | * | * | * |  | * | * | * | * | 7 |
| Sun et al | * | * | * | * | * |  | * |  | 6 |
| Zeng et al | * | * | * | * | * | * | * | * | 8 |
| Zhao et al | * | * | * | * | * |  | * |  | 6 |
| Wu et al | * | * | * | * | * | * | * | * | 8 |
| Cao et al | * | * | * | * | * | * | * |  | 7 |
| Chaib et al | * | * | * | * | * |  |  |  | 6 |
| Chen et al | * | * | * | * | * | * | * |  | 7 |
| Gao et al | * | * | * | * | * |  | * |  | 6 |
| Hong et al | * | * | * | * | * |  | * |  | 6 |
| Huang et al | * | * | * | * | * |  | * |  | 6 |
| Liu^1^ et al | * | * | * | * | * | * | * |  | 6 |
| Liu^2^ et al | * | * | * | * | * |  | * | * | 7 |
| Liu^3^ et al | * | * | * | * | * | * | * |  | 7 |
| Pan et al | * | * | * | * | * | * | * |  | 7 |
| Rybarczyk et al | * | * | * | * | * |  | * |  | 6 |
| Sugimachi et al | * | * | * | * | * | * | * | * | 8 |
| Wei et al | * | * | * | * | * |  | * |  | 6 |
| Wu et al | * | * | * | * | * | * | * |  | 7 |
| Zhang et al | * | * | * | * | * |  | * |  | 6 |
| Chen et al | * | * | * | * | * | * | * |  | 7 |
| Ding et al | * | * | * | * | * | * | * |  | 7 |
| Godlewski et al | * | * | * | * | * | * | * |  | 7 |
| Guichet et al | * | * | * | * | * | * | * |  | 7 |
| Hong et al | * | * | * | * | * | * | * |  | 7 |
| Huang et al | * | * | * | * | * |  | * |  | 6 |
|  |  |  |  |  |  |  |  |  |  |
| Kim et al | * | * | * | * | * | * | * |  | 7 |
| Luu et al | * | * | * | * | * | * | * |  | 7 |
| Qian et al | * | * | * | * | * | * | * |  | 7 |
| Chen et al | * | * | * | * | * | * | * |  | 7 |
| Kim et al | * | * | * | * | * | * | * |  | 7 |
| Van Haele et al | * | * | * | * | * | * | * |  | 7 |
| Zhang et al | * | * | * | * | * | * | * | * | 8 |

Abbreviation: YAP1 yes-associated protein 1; NOS Newcastle-Ottawa Scale.
