# Supplementary material for: The prognostic value of YAP1 on clinical outcomes in human cancers
Source: Aging (Albany NY). 2019 Oct 15;11(19):8681–700. doi: 10.18632/aging.102358 (PMC6814621; doi:10.18632/aging.102358)
Supplement: Supplementary Table 3 [file aging-11-102358-s003.docx]

**Supplementary Table 3. Characteristics of Meta-analysis.**

| **First author** | **Year** | **Patient source** | **No of patient** | **Tumor type** | **Specimen** | **Method** | **YAP1 Expression(%)** | **Staining location** | **Median (range) follow-up**  **(month)** | **Outcome** | **Analysis** | **HR estimation** | **HR(95%CI)** | **NOS score** |
| --- | --- | --- | --- | --- | --- | --- | --- | --- | --- | --- | --- | --- | --- | --- |
| Xu et al[[27](#_ENREF_25)] | 2009 | China | 176 | Liver cancer | Tissue | IHC | Positive:109(61.9%) | YAP1 expression | NA | OS | M | Reported in text | 2.148(1.255-3.677) | 6 |
|  |  |  |  |  |  |  | Positive:102(61.4%) |  |  | DFS |  |  | 1.653(1.081-2.528) |  |
| Hall et al[[28](#_ENREF_26)] | 2010 | America | 67 | Ovarian cancer | Tissue | IHC | High:25(35.7%) | Nuclear YAP1 expression | NA | DSS | U | Reported in text | 3.3(1.03-10.9) | 7 |
| Wang^1^ et al[[30](#_ENREF_28)] | 2010 | China | 95 | Lung cancer | Tissue | IHC | Positive:53(55.8%) | YAP1 expression | NA | OS | U | Data extrapolated | 2.31(1.41-3.79) | 7 |
| Wang^2^ et al[[29](#_ENREF_27)] | 2010 | China | 100 | Gastric cancer | Tissue | IHC | Positive:61(61.0%) | YAP1 expression | NA | OS | U | Data extrapolated | 1.04(0.34-3.18) | 8 |
|  |  |  |  |  |  |  |  |  |  | DFS |  |  | 1.09(0.52-2.29) |  |
| Kang et al[[31](#_ENREF_29)] | 2011 | China | 129 | Gastric cancer | Tissue | IHC | High:94(72.0%) | Nuclear YAP1 expression | 17.2(0.3-143.4) | DSS | U | Data extrapolated | 1.64(0.90-2.90) | 6 |
| Muramatsu et al[[18](#_ENREF_16)] | 2011 | Japan | 120 | Esophageal cancer | Tissue | IHC | Positive:69(57.5%) | Nuclear YAP1 expression | 19 (1-103) | OS | U | Reported in text | 1.764(1.081-2.882) | 7 |
|  |  |  |  |  |  |  | Positive:74(61.7%) | Cytoplasmic YAP1 expression |  | OS | U | Data extrapolated | 1.10(0.66-1.86) |  |
| Song et al[[32](#_ENREF_30)] | 2012 | Korea | 223 | Gastric Cancer | Tissue | IHC | Positive:61(27.4%) | Nuclear YAP1 expression | NA | OS | U | Reported in text | 1.233(0.789-1.927) | 7 |
| Yeo et al[[33](#_ENREF_31)] | 2012 | Korea | 142 | Esophageal cancer | Tissue | IHC | High:38(26.8%) | Nuclear YAP1 expression | NA | OS | U | Data extrapolated | 2.43(1.45-4.09) | 7 |
|  |  |  |  |  |  |  |  |  |  | DFS |  |  | 2.04(1.3-3.2) |  |
| Kim^1^ et al[[35](#_ENREF_33)] | 2013 | Korea | 153 | Liver cancer | Tissue | IHC | Positive:67(43.8%) | YAP1 expression | 32.8 (4.3-128.7) | OS | U | Reported in text | 1.99(0.988-4.008) | 8 |
|  |  |  |  |  |  |  |  |  |  | DFS |  |  | 1.261(0.777-2.046) |  |
| Kim^2^ et al[[34](#_ENREF_32)] | 2013 | Korea | 132 | Colorectal cancer | Tissue | IHC | Positive:48(36.4%) | Nuclear YAP1 expression | NA | OS | M | Reported in text | 1.206(0.606-2.4) | 6 |
| Liu^1^ et al[[36](#_ENREF_34)] | 2013 | China | 213 | Bladder cancer | Tissue | IHC | Positive:113(53.1%) | YAP1 expression | NA | OS | M | Reported in text | 3.553(1.561-8.086) | 7 |
| Liu^2^ et al[[37](#_ENREF_35)] | 2013 | China | 42 | Cervical cancer | Tissue | IHC | High:11(26.2%) | Nuclear YAP1 expression | 74.5（10-113） | OS | U | Data extrapolated | 2.08(0.4-10.96) | 7 |
|  |  |  |  |  |  |  |  |  |  | DFS |  |  | 1.45(0.13-16.23) |  |
| Xu^1^ et al[[40](#_ENREF_38)] | 2013 | China | 80 | Liver cancer | Tissue | IHC | Positive:33(41.2%) | YAP1 expression | NA | OS | U | Data extrapolated | 4.74(1.5-15) | 6 |
|  |  |  |  |  |  |  |  |  |  | RFS |  |  | 5.41(2.95-9.95) |  |
| Xu^2^ et al[[41](#_ENREF_39)] | 2013 | China | 227 | Liver cancer | Tissue | IHC | Positive:129(56.8%) | YAP1 expression | NA | OS | M | Reported in text | 1.569(1.008-2.441) | 6 |
|  |  |  |  |  |  |  |  |  |  | DFS |  |  | 1.489(1.011-2.193) |  |
| Wang^1^ et al[[39](#_ENREF_37)] | 2013 | China | 139 | Colorectal cancer | Tissue | IHC | High:73(52.5%) | YAP1 expression | NA | OS | M | Reported in text | 1.907(1.104-3.293) | 6 |
| Wang^2^ et al[[38](#_ENREF_36)] | 2013 | China | 168 | Colorectal cancer | Tissue | IHC | Positive:122(72.6%) | YAP1 expression | 43(1-56) | OS | M | Reported in text | 2.168(1.125-4.179) | 7 |
| Hu et al[[42](#_ENREF_40)] | 2014 | China | 191 | Gastric cancer | Tissue | IHC | Positvie:147(68.7%) | YAP1 expression | NA | DFS | M | Reported in text | 1.167(1.017-2.762) | 7 |
| Kim^1^ et al[[44](#_ENREF_42)] | 2014 | Korea | 678 | Breast cancer | Tissue | IHC | Positive:42(6.2%) | Nuclear YAP1 expression | NA | OS | M | Reported in text | 2.073(0.973-4.416) | 6 |
|  |  |  |  |  |  |  |  |  |  | DFS |  |  | 1.867(0.841-4.147) |  |
| Kim^2^ et al[[43](#_ENREF_41)] | 2014 | Korea | 183 | Breast cancer | Tissue | IHC | Positive:18(9.8%) | YAP1 expression | NA | OS | M | Reported in text | 1.314(0.161-10.733) | 6 |
|  |  |  |  |  |  |  |  |  |  | DFS |  |  | 3.206(1.00-10.27) |  |
| Sun et al[21] | 2014 | Korea | 205 | Lung cancer | Tissue | IHC | High:116(56.6%) | Cytoplasmic YAP1 expression | NA | OS | M | Reported in text | 0.474(0.263-0.854) | 7 |
|  |  |  |  |  |  |  |  |  |  | PFS |  |  | 0.659(0.431-1.01) |  |
| Tsujiura et al[[46](#_ENREF_44)] | 2014 | America | 120 | Endometrial cancer | Tissue | IHC | High:66(55.0%) | Nuclear YAP1 expression | NA | OS | U | Data extrapolated | 0.12(0.01-3.83) | 7 |
| Xia et al[[47](#_ENREF_45)] | 2014 | China | 45 | Ovarian cancer | Tissue | IHC | Positive:33(73.3%) | Nuclear YAP1 expression | NA | DSS | U | Data extrapolated | 1(0.39-2.57) | 6 |
|  |  |  |  |  |  |  |  | YAP1 expression |  |  |  |  | 1.65(0.65-4.19) |  |
| Ahmed et al[[48](#_ENREF_46)] | 2015 | America | 32 | Ewing’s sarcoma | Tissue | IHC | High:10(31.2%) | YAP1 expression | NA | DFS | U | Reported in text | 0.9(0.295-2.916) | 7 |
| Hayashi et al[[49](#_ENREF_47)] | 2015 | Japan | 95 | Liver cancer | Tissue | PCR | High:24(25.3%) | YAP1 expression | NA | OS | U | Data extrapolated | 2.95（0.93-9.41） | 7 |
| Kim^1^ et al[[50](#_ENREF_48)] | 2015 | Korea | 122 | Breast cancer | Tissue | IHC | Positive:7(5.7%) | Nuclear YAP1 expression | NA | OS | U | Data extrapolated | 3.73(1.45-9.63) | 7 |
| Kim^2^ et al[[51](#_ENREF_49)] | 2015 | Korea | 167 | Lung cancer | Tissue | IHC | High:34(20.4%) | Nuclear YAP1 expression | 22 | DFS | U | Data extrapolated | 2.1(0.93-4.76) | 6 |
| Li et al[[45](#_ENREF_43)] | 2015 | China | 105 | Liver cancer | Tissue | IHC | Positive:54(51.4%) | YAP1 expression | 13(1-96) | DFS | M | Reported in text | 2.011(1.115-3.628) | 7 |
| Liu et al[[52](#_ENREF_50)] | 2015 | China | 75 | Ovarian cancer | Tissue | IHC | High:20(26.7%) | Nuclear YAP1 expression | NA | OS | U | Data extrapolated | 0.98(0.38-2.49) | 6 |
|  |  |  |  |  |  |  | High:51(68%) | Cytoplasmic YAP1 expression |  |  |  |  | 1.45(0.64-3.31) |  |
| Pei et al[[53](#_ENREF_51)] | 2015 | China | 90 | Liver cancer | Tissue | IHC | High:39(43.3%) | YAP1 expression | NA | OS | M | Reported in text | 1.742(1.067-2.844) | 7 |
| Suh et al[[54](#_ENREF_52)] | 2015 | Korea | 116 | Gastric cancer | Tissue | IHC | Positive: 50(43.1%) | YAP1 expression | NA | OS | U | Data extrapolated | 0.34(0.04-2.8) | 6 |
|  |  |  |  |  |  |  |  |  |  | DFS |  |  | 0.5(0.13-1.95) |  |
| Wang et al[[55](#_ENREF_53)] | 2015 | China | 95 | Liver cancer | Tissue | IHC | High:53(55.8%) | YAP1 expression | NA | OS | U | Data extrapolated | 2.44(1.46-4.09) | 8 |
| Abduch et al[[56](#_ENREF_54)] | 2016 | Brazil | 31 | Adrenocortical tumors | Tissue | PCR | High:11(35.5%) | YAP1 expression | 42(3.6-211.2) | OS | U | Data extrapolated | 2.02(0.35-11.62) | 7 |
| Cao et al[[57](#_ENREF_55)] | 2016 | China | 82 | Colorectal cancer | Tissue | IHC | High:32(39%) | YAP1 expression | NA | OS | U | Data extrapolated | 2.18(1.02-4.63) | 8 |
| Ni et al[61] | 2016 | China | 86 | Ovarian cancer | Tissue | IHC | High:53(61.6%) | YAP1 expression | NA | OS | U | Data extrapolated | 1.9(0.77-4.68) | 6 |
| Li^1^ et al[59] | 2016 | China | 161 | Gastric cancer | Tissue | IHC | High:132(82%) | YAP1 expression | NA | OS | M | Reported in text | 2.506(1.195-5.257) | 7 |
| Li^2^ et al[58] | 2016 | China | 46 | Colorectal cancer | Tissue | IHC | Positive:32(69.6%) | YAP1 expression | 16(2-30) | DFS | U | Data extrapolated | 0.84(0.18-3.9) | 7 |
| Luo et al[[60](#_ENREF_58)] | 2016 | China | 62 | Ovarian cancer | Tissue | IHC | Positive:54(87.1%) | YAP1 expression | 31.5(6-70.5) | PFS | U | Data extrapolated | 1.65(0.23-12.00) | 7 |
| Sun et al[[62](#_ENREF_60)] | 2016 | China | 270 | Gastric cancer | Tissue | IHC | High:210(77.8%) | YAP1 expression | 51.8 | OS | M | Reported in text | 0.794(0.563-1.121) | 6 |
| Zhao et al[[64](#_ENREF_62)] | 2016 | China | 81 | Lung cancer | Tissue | IHC | High:46(56.8%) | YAP1 expression | NA | OS | U | Data extrapolated | 2.14(1.00-4.57) | 6 |
| Wu et al[[63](#_ENREF_61)] | 2016 | China | 137 | Liver cancer | Tissue | IHC | High:78(56.9%) | YAP1 expression | 40(6-142) | RFS | M | Reported in text | 2.303(1.142-3.375) | 8 |
|  |  |  |  |  |  |  |  |  |  | DSS |  |  | 2.236(1.374-3.64) |  |
|  |  |  | 122 |  |  |  | High:82(67.2%) |  |  | RFS |  |  | 2.161(1.3-3.593) |  |
|  |  |  |  |  |  |  |  |  |  | DSS |  |  | 2.134(1.288-3.536) |  |
| Cao et al[[65](#_ENREF_63)] | 2017 | China | 184 | Breast cancer | Tissue | IHC | Positive:134(53.4%) | YAP1 expression | NA | DFS | M | Reported in text | 0.547(0.32-0.934) | 7 |
| Chaib et al[[66](#_ENREF_64)] | 2017 | Spain,Italy,France,Colombia | 64 | Lung cancer | Tissue | PCR | NA | YAP1 expression | NA | PFS | U | Reported in text | 2.57(1.3-5.09) | 6 |
|  |  | China | 55 |  |  |  |  |  |  |  |  |  | 2.53(1.37-4.66) |  |
| Chen et al[[67](#_ENREF_65)] | 2017 | China | 55 | Bladder cancer | Tissue | IHC | Positive:27(49.1%) | YAP1 expression | NA | OS | U | Data extrapolated | 2.85(1.19-6.83) | 7 |
| Gao et al[[68](#_ENREF_66)] | 2017 | China | 75 | Ovarian cancer | Tissue | IHC | High：20(26.7%) | Nuclear YAP1 expression | NA | OS | U | Data extrapolated | 1.68(0.72-3.91) | 6 |
| Hong et al[[69](#_ENREF_67)] | 2017 | Korea | 166 | Gastric cancer | Tissue | IHC | High:81(48.8%） | Nuclear YAP1 expression | NA | OS | U | Data extrapolated | 1.31(0.84-2.03) | 6 |
|  |  |  |  |  |  |  |  |  |  | DFS |  |  | 1.24(0.82-1.87) |  |
| Huang et al[[70](#_ENREF_68)] | 2017 | China | 120 | Gastric cancer | Tissue | IHC | Positive:58（48.3%） | Nuclear YAP1 expression | NA | OS | M | Reported in text | 5.113(2.123-12.316) | 6 |
| Liu^1^ et al[[72](#_ENREF_70)] | 2017 | China | 72 | Glioma | Tissue | IHC | High:51(70.8%) | YAP1 expression | NA | OS | U | Reported in text | 1.139(1.074-1.207) | 6 |
| Liu^2^ et al[[71](#_ENREF_69)] | 2017 | China | 138 | Liver cancer | Tissue | IHC | High:66(47.8%) | YAP1 expression | NA | OS | M | Reported in text | 1.533(1.059-2.219) | 7 |
|  |  |  |  |  |  |  |  |  |  | RFS |  |  | 1.474(1.029-2.112) |  |
| Liu^3^ et al[[73](#_ENREF_71)] | 2017 | China | 240 | Thyroid cancer | Tissue | IHC | Positive:149(62.1%) | Nuclear YAP1 expression | NA | RFS | M | Reported in text | 4.878(1.082-21.739) | 7 |
| Pan et al[[74](#_ENREF_72)] | 2017 | China | 91 | Laryngeal carcinoma | Tissue | IHC | Positive:56(61.5%) | YAP1 expression | NA | OS | U | Reported in text | 4.52(1.83-6.65) | 7 |
| Rybarczyk et al[[75](#_ENREF_73)] | 2017 | Poland | 86 | Renal cancer | Tissue | PCR | High:60(69.8%) | YAP1 expression | NA | OS | M | Reported in text | 4.03(0.96-16.79) | 6 |
|  |  |  |  |  |  |  | High:33(38.2%) |  |  | PFS |  |  | 1.84(0.45-6.23) |  |
| Sugimachi et al[[76](#_ENREF_74)] | 2017 | Japan | 88 | Liver cancer | Tissue | IHC | High:28(31.8%) | Nuclear YAP1 expression | 20(1-182) | OS | U | Data extrapolated | 2.23(1.21-4.11) | 8 |
| Wei et al[[77](#_ENREF_75)] | 2017 | China | 63 | Pancreatic cancer | Tissue | IHC | High:37(58.7%) | Nuclear YAP1 expression | NA | OS | U | Data extrapolated | 0.67(0.07-6.44) | 6 |
| Wu et al[[78](#_ENREF_76)] | 2017 | Taiwan | 85 | Colorectal cancer | Tissue | IHC | High:41(48.2%) | YAP1 expression | NA | OS | M | Reported in text | 3.4(1.69-6.86) | 7 |
|  |  |  |  |  |  |  |  |  |  | RFS |  |  | 2.68(1.45-4.96) |  |
| Zhang et al[[79](#_ENREF_77)] | 2017 | China | 142 | Esophageal cancer | Tissue | IHC | Positive:124(87.3%) | YAP1 expression | NA | OS | M | Reported in text | 2.78(0.958-8.083) | 6 |
| Chen et al[[80](#_ENREF_78)] | 2018 | China | 114 | Liver cancer | Tissue | IHC | Positive:71(62.3%) | YAP1 expression | NA | DFS | U | Reported in text | 1.82(1.06-3.10) | 7 |
| Godlewski et al[[81](#_ENREF_79)] | 2018 | Poland | 54 | Renal cancer | Tissue | IHC | Positive:35(64.8%) | Nuclear YAP1 expression | 40.6 | OS | U | Reported in text | 0.63(0.38-1.02) | 7 |
|  |  |  |  |  |  |  | Positive:41(64.8%) | Cytoplasmic YAP1 expression |  |  | M |  | 4.53 (1.73-11.9) |  |
| Guichet et al[[82](#_ENREF_80)] | 2018 | France | 117 | Glioma | Tissue | IHC | High:52(44.4%) | YAP1 expression | NA | OS | M | Reported in text | 2.615(1.181 - 5.79) | 7 |
|  |  |  |  |  |  |  |  |  |  | PFS |  |  | 1.501(0.761-2.959) |  |
| Hong et al[[83](#_ENREF_81)] | 2018 | Korea | 63 | Lung cancer | Tissue | IHC | High:25(39.7%) | Nuclear YAP1 expression | 33.9(25.4-42.4) | OS | U | Data extrapolated | 2.05(0.97-4.31) | 7 |
|  |  |  |  |  |  |  |  |  | 12.5(8.2–16.8) | PFS |  |  | 1.95(0.85-4.49) |  |
| Huang et al[[84](#_ENREF_82)] | 2018 | China | 62 | Gallbladder carcinoma | Tissue | IHC | Positive:40(64. 5%) | YAP1 expression | NA | OS | U | Data extrapolated | 1.46(0.65-3.26) | 6 |
| Kim et al[[85](#_ENREF_83)] | 2018 | Korea | 50 | Liver cancer | Tissue | IHC | High:19(38%) | Nuclear YAP1 expression | 56.7(1.1-137.7) | OS | U | Data extrapolated | 2.64(0.68-10.28) | 7 |
|  |  |  |  |  |  |  |  |  |  | RFS |  |  | 2.73(1.03-7.24) |  |
| Luu et al[[86](#_ENREF_84)] | 2018 | Canada | 11 | Osteosarcoma | Tissue | IHC | High:9(81.8%) | YAP1 expression | NA | OS | U | Data extrapolated | 0.90(0.11-7.28) | 7 |
| Qian et al[[87](#_ENREF_85)] | 2018 | China | 66 | Colorectal cancer | Tissue | IHC | High:36(54.5%) | YAP1 expression | NA | OS | U | Data extrapolated | 1.80(0.83-3.92) | 7 |
| Chen et al[[88](#_ENREF_87)] | 2019 | Taiwan | 102 | Lung cancer | Tissue | IHC | High:45(44.1%) | Nuclear YAP1 expression | 26.3(1-165.3) | OS | M | Reported in text | 1.89(1.14-3.13) | 7 |
|  |  |  |  |  |  |  |  |  |  | RFS |  |  | 1.73(1.06-2.83) |  |
| Ding et al[[89](#_ENREF_88)] | 2019 | China | 110 | Breast cancer | Tissue | IHC | High:51(46.4%) | YAP1 expression | 22(2-106) | DFS | U | Data extrapolated | 1.26(0.82-1.93) | 7 |
|  |  |  | 48 |  |  |  | High:22(45.8%) | Nuclear YAP1 expression |  | DFS | M | Reported in text | 3.199(1.489-6.874) |  |
|  |  |  |  |  |  |  | High:14(29.2%) | Cytoplasmic YAP1 expression |  | DFS | M | Reported in text | 0.923(0.425-2.008) |  |
| Kim et al[[90](#_ENREF_89)] | 2019 | Korea | 318 | Gastric cancer | Tissue | IHC | High:108(34.0%) | YAP1 expression | 43(1-99) | OS | M | Reported in text | 2.938(1.726-4.998) | 7 |
| Van Haele et al[[91](#_ENREF_90)] | 2019 | Belgium | 81 | Liver cancer | Tissue | IHC | Positive:52(64.2%) | YAP1 expression | NA | OS | M | Reported in text | 2.823 (0.946-8.424) | 7 |
|  |  |  |  |  |  |  |  |  |  | DFS |  |  | 1.705 (0.578-5.028) |  |
|  |  |  |  |  |  |  |  | Cytoplasmic YAP1 expression |  | OS |  |  | 4.395 (1.235-15.637) |  |
|  |  |  |  |  |  |  |  |  |  | DFS |  |  | 2.622 (0.759-9.054) |  |
|  |  |  |  |  |  |  |  | Nuclear YAP1 expression |  | OS | U |  | 1.212 (0.583-2.519) |  |
|  |  |  |  |  |  |  |  |  |  | DFS |  |  | 1.093 (0.527-2.267) |  |
| Zhang et al[[92](#_ENREF_91)] | 2019 | China | 166 | Colorectal cancer | Tissue | IHC | Positive:131(78.9%) | YAP1 expression | 129(5-174) | OS | M | Reported in text | 0.25(0.14-0.46) | 8 |

Abbreviations: *CI* confidence interval; *DFS* disease-free survival; *DSS* disease-specific survival; *HR* hazard ratio; *IHC* immunohistochemistry; *M* multivariate; *NA* no applicable; *NOS* New-Ottawa Scale; *OS* overall survival; *PFS* progression-free survival; *RFS* recurrence-free survival; *U* univariate.
